# Benchmarking Australian Dental Weighted Activity Units against public and private dental fee schedules: a cross-sectional item-code comparator study

**DOI:** 10.1038/s41405-026-00479-0

**Published:** 2026-08-18

**Authors:** Jonathon Oddy, William Ha

**Affiliations:** Faculty of Medicine and Health—School of Dentistry, The University of Sydney, Sydney, NSW Australia

**Keywords:** Alcohol misuse in dental patients, Dental business

## Abstract

**Objective:**

To benchmark Dental Weighted Activity Units (DWAUs) against Australian dental remuneration schedules and derive suggested recalibration factors for endodontics, periodontics and prosthodontics.

**Materials and methods:**

A cross-sectional item-code comparator study linked Australian Dental Association item codes to DWAU values and fees from public and private schedules, standardised to 1 January 2025 Australian dollars. The primary outcome was grouped DWAU per dollar, calculated as the sum of DWAUs divided by the sum of fees and expressed as DWAU per $1000. Lower values indicated fewer DWAUs credited per dollar of remuneration. Merged general and merged specialist fee series were constructed, with bootstrap 95% confidence intervals and suggested stream-specific calibration factors estimated.

**Results:**

Of 343 item codes, 264 had DWAU values greater than zero and 260 were included. Overall DWAU per $1000 was 1.589 for merged general comparators and 1.130 for merged specialist comparators. Under specialist comparators, endodontics had the lowest value at 0.945 DWAU per $1000, 16.40% below the benchmark. The suggested specialist calibration factor for endodontics was 1.196.

**Discussion:**

The findings indicate that current DWAU values are not uniformly aligned with remuneration signals, particularly for specialist endodontics, and supports stream-specific review rather than a single uniform adjustment.

**Conclusions:**

Cross-schedule benchmarking and specialist-adjusted DWAU rates may improve public dental activity measurement, commissioning and specialist service planning.

## Introduction

The Australian dental healthcare system operates under multiple public and private funding models. Medicare covers all Australians for medical services, but it does not cover dental care [1]. The current system has distinct reimbursement structures and implementations for general dental and specialist care services. Public dental healthcare is primarily funded and managed by state and territory governments. However, the federal government also provides financial support through specific programmes [2]. Public dental services are generally available to eligible patients including concession card holders and children, with free or limited co-payments to the individual [3–10]. The federal government provides funding through the Child Dental Benefits Schedule (CDBS) for children aged 17 years or younger, through the Department of Veterans’ Affairs (DVA) for eligible veterans and through financial grants to state governments under the National Partnership Agreement [11–13].

Public funding is generally structured around fee schedules or activity-based funding using the Dental Weighted Activity Unit (DWAU) system [14]. DWAUs are administrative weighting units used to quantify relative dental activity and support funding allocation. These determine valuation rates based on the complexity of treatment and resource requirements.

The DWAU system was introduced through the 2012 National Partnership Agreement and is used as a standardised public dental valuation model [15]. As part of the agreement, the federal government agreed to provide annual monetary grants of $107.75 million to the states collectively if key clinical milestones were achieved [13]. The DWAU system is used as a standardised valuation model within public dental healthcare to effectively assess total treatment outputs. The unit-based system assigns weightings to various dental procedures depending on their complexity, time and resource requirements [15]. The DWAU system has been criticised for failing to accurately reflect the true cost and resource intensity of certain specialist procedures as well as the ongoing impacts of COVID-19 on the health sector [16].

DWAU allocations are functionally static and do not adjust to reflect changing treatment methods, equipment or specialist skills.

Approximately two thirds of the Australian population is not covered under a Commonwealth or state funded dental programme and must bear the costs of dental care independently or through health insurance funds [17]. These private dental services operate on a market-driven, fee-for-service model. This approach allows specialists to set their prices according to overhead costs, expertise and patient demand. Private health insurance plays a role in subsidising specialist fees, however, the amount reimbursed is dependent on numerous factors such as the healthcare fund and preferred provider status [18].

In 2024, 54.9% of Australians had private general treatment extras coverage which generally covers dental services [19]. However, people with private health insurance still face significant costs for their dental care with 54% of treatment costs being reimbursed by private insurance funds [17]. Some insurers operate preferred provider networks, where specialists offer services at pre-negotiated rates [18]. These networks allow insurers to set fixed reimbursement rates which can reduce out-of-pocket expenses for the patient, however, this does limit the choice of provider [18].

DVA offers fully paid dental treatments for eligible veterans through private sector clinicians. However, the fee schedule for DVA patients was shown to be on average 21% lower than the averaged private fee schedule [20]. Additionally, clinicians treating DVA-funded patients are not permitted to charge a gap fee to adjust the total remuneration for treatment [21]. This has led to a growing gap between private treatment costs and the fees paid under the DVA schedule [22]. As a result, veterans may have difficulty accessing healthcare as there is a limited pool of clinicians who accept DVA patients [22].

The Australian Defence Force (ADF) provides healthcare to its serving members either through direct care services or via the health services contract with Bupa [23]. This contract was introduced in 2019 and facilitates ADF personnel to access private specialist dental services, with their treatment costs covered by the ADF at fixed specialist rates [23, 24]. This model operates independently of mainstream public funding structures, limiting its applicability to civilian healthcare settings. However, it does provide an example of a fee rate that is generally accepted by private specialists and funded by public resources.

The CDBS, introduced by the Australian Government in 2014, provides eligible children aged 0–17 with access to dental healthcare through public or private clinicians. In 2021, 51.1% of all children were eligible under the CDBS [25]. It replaced the Medicare Teen Dental Plan and aimed to improve oral health outcomes for children by covering up to $1132.00 per eligible child over two calendar years [17]. These funds can be used for treatment provided by general and specialist dentists in both private and public settings [11]. Approximately 83% of services have been delivered via private clinicians and 17% by state public clinics [26]. While these private clinicians are not required to be bulk billed, they almost always do with bulk billing rates at 95% [26].

The Oral Health Fee for Service Scheme (OHFFSS) operates in New South Wales as a mechanism to allow approved public patients to have dental care provided by private practitioners [27]. It is generally used as part of a voucher system with maximum reimbursement limits per treatment. For example, the ‘Urgent Care Voucher’ provides up to $480.00 [28]. Treatment provided under this system follows a fee-for-service model in which reimbursement rates are set per ADA item code [27, 28].

In the public sector, fee schedules and DWAU allocations can be misaligned for different treatments and fields of specialist dentistry. Specialist fields such as prosthodontics, periodontics and endodontics are part of the public system and reviewing their DWAUs in comparison to other remuneration and valuation systems will provide additional insights into the suitability and efficacy of the current DWAUs.

Disparities in public funding also have broader economic implications. Inadequate public funding forces dental clinics to operate under financial constraints, limiting their ability to expand specialist services or invest in new technologies. Patients relying on public services often experience extended wait times due to funding shortages and resource limitations, especially for specialist services. In New South Wales during 2022-23, the waitlist for general dental services averaged 387 days [2].

In contrast, private sector patients enjoy faster access to care, albeit with higher financial costs.

Overall, Australia’s specialist dental funding landscape is fragmented, featuring multiple reimbursement models with varying fee structures and payment mechanisms. The significant differences between DWAUs, private fees, health insurance rebates and government fee-for-service models create a complex funding environment. This study compares these models to identify trends and potential discrepancies in the current system.

Australia uses a single national list of ADA item codes to describe both general and specialist dental treatment. Public dental activity is quantified through DWAU values attached to that single list, and there is no separate specialist DWAU schedule. Multiple public and private fee schedules operate in parallel over the same item codes, each with its own purpose and payment rules. The distinction between general and specialist care in this study is therefore made through the comparator fee schedules used, rather than through separate code lists or weighting schedules.

## Materials and methods

### Study design

This study used a cross-sectional observational comparator design to examine the relationship between DWAUs and remuneration across Australian dental fee schedules. The unit of analysis was the ADA item code, treated as a single observation linked to its DWAU value and the comparator fees available for that code. The aim was to assess whether the DWAU system credits output evenly per dollar of external remuneration across specialist streams, which would indicate whether the current DWAU scale is aligned with prevailing remuneration signals.

A single national list of ADA item codes, with one DWAU value per code, applies to both general and specialist dental care; the DWAU schedule itself does not distinguish between provider types. The distinction between general and specialist streams in this study therefore arises solely from the comparator fee schedules used to calculate DWAU per dollar, and not from separate general and specialist DWAU code lists. Throughout this article, the term ‘stream’ refers to a clinical discipline grouping of item codes (endodontics, periodontics or prosthodontics) and does not denote a continuous flow of data in the statistical sense, while the term ‘series’ refers to a merged comparator fee dataset constructed at item-code level (the merged general series or the merged specialist series).

The main item-level metric was DWAU per dollar (DWAU/$), calculated as DWAU divided by the comparator fee for each code. Item-level DWAU/$ values were used descriptively and to support derivation of the suggested calibration factors. The primary statistical summary was grouped DWAU/$. This was defined as the sum of DWAUs divided by the sum of fees (ΣDWAUs ÷ Σfees) within the relevant grouping (overall or by specialist stream), rather than the mean of code-level ratios. This approach was used because it reflects the overall DWAU credit awarded per dollar of remuneration for the group and reduces distortion from small-fee or small-DWAU item codes with outlier ratios.

For this study, the codes were classified into three streams: endodontics, periodontics and prosthodontics. These three streams were selected because they represent the main specialist disciplines of interest and have sufficient item-code coverage across the comparator schedules for meaningful analysis. Codes were assigned to streams by the authors using the service-category groupings and item descriptors of the ADA Schedule (for example, endodontic items within the 400-series), with the mapping cross-checked against schedule descriptors and finalised by consensus before analysis. Simple procedures that may be provided by specialists but are not discipline-specific (for example, removal of calculus) were not assigned to a specialist stream; such codes contributed to the overall benchmark for each merged series, which was calculated across all eligible codes, but not to the stream-level estimates, which comprised discipline-specific codes only.

Grouped DWAU/$ outputs were also used to derive suggested calibration factors. These factors define two illustrative calibrated schedules: one aligned to the merged general benchmark and one aligned to the merged specialist benchmark. The specialist-aligned approach was used to derive a suggested specialist DWAU rate intended to reflect specialist remuneration patterns more directly than the existing general DWAU scale. Neither schedule replaces the published DWAU values; both are presented for consideration only.

The study was reported with reference to the Strengthening the Reporting of Observational Studies in Epidemiology (STROBE) guidance for observational studies.

### Data sources and variables

Activity weights were obtained from the relevant DWAU schedule [13–15]. Fee data were drawn from multiple sources to capture general and specialist remuneration across public and private settings. Survey-based general and specialist fees were obtained from the ADA Dental Fees Survey. For the ADA fee survey, jurisdictional variation was handled using a national mean approach [29]. Additional comparators were obtained from the DVA fee schedule (1 January 2025 values) and the CDBS for 1 January 2025 to 31 December 2025 [11, 12, 30]. Where included, the OHFFSS contributed voucher-based rates for general services [28]. Aggregated private specialist fee schedules were incorporated for the specified period. Private health insurance (PHI) typical price data were included for the limited subset of item codes with PHI values and were deflated by 6 months to the 01 January 2025 baseline using a compounded pro-rata adjustment [31].

Key extracted variables included DWAU values and item descriptors from the DWAU schedule, item-level fees from the ADA, ADF, DVA, PHI and CDBS sources, and code-level private specialist fees from de-identified aggregated schedules. Scheme-specific rules relevant to interpretation (for example, caps or voucher limits) were documented separately and used to inform inclusion decisions and comparability across sources.

### Eligibility criteria

Item codes were eligible if they had a valid DWAU value greater than zero and at least one comparator fee available in either the merged general or merged specialist fee series. Codes were mapped to endodontics, periodontics and prosthodontics using a pre-specified stream mapping approach.

Codes were excluded if DWAU was missing or equal to zero, if an item was not comparable across schedules due to unresolved definitional differences, or if the charging unit could not be resolved from schedule descriptors. Exclusions were documented.

### Variables and derivations

For each item code, the core variables were item code, item description, assigned stream and DWAU value. Comparator fees were extracted and standardised to a common price date. Two merged fee series were derived at item-code level:

- **Merged general fee:** Mean of available general-dentist comparator sources for that code.
- **Merged specialist fee:** Mean of available specialist comparator sources for that code.

The merged general fee series included available general comparator sources, including ADA general, ADF general, DVA general, CDBS, PHI typical prices and OHFFSS where item-level matches were available. OHFFSS was included as an additional general comparator fee source (not used in the specialist series). The merged specialist fee series included ADA specialist, ADF specialist, DVA specialist, PHI typical prices and aggregated private specialist fees where available.

All monetary values were expressed in Australian dollars standardised to 01 January 2025. Fee sources with effective dates before this baseline were uplifted using the Australian Bureau of Statistics All Groups Consumer Price Index (CPI) according to [32, 33]:

The CPI series and factor applied to each source were stored with the adjusted series for auditability. ADA and ADF values (effective 01 July 2024) were uplifted to the baseline, while CDBS and DVA values commencing 01 January 2025 were treated as unadjusted. Private health insurance typical price values were additionally deflated by 6 months to the baseline using a compounded pro-rata CPI adjustment.

### Derivation of suggested calibration factors

Published DWAU values were not adjusted before analysis; all primary results are reported using unadjusted DWAU values, and the factors described in this section are suggested calibration factors presented for consideration rather than adjustments applied prior to inference. With the proposed general calibration, stream multipliers were derived so that each stream’s grouped DWAU per dollar (DWAU/$) would align to the overall grouped DWAU/$ benchmark in the merged general series. The overall grouped DWAU/$ benchmark for each merged series was calculated across all eligible codes included in that merged series, not only the three specialist streams. Streams with lower DWAU/$ than the benchmark (fewer DWAUs credited per dollar) were assigned an upward suggested calibration factor, while streams with higher DWAU/$ than the benchmark were assigned a downward suggested calibration factor.

With the specialist calibration, the same approach was applied using the merged specialist benchmark. The suggested factors were derived for endodontics, periodontics and prosthodontics to define a specialist-aligned DWAU schedule and a suggested specialist DWAU rate. The effects of the suggested calibration factors were assessed by reporting grouped DWAU/$ by stream before and after applying the factors within the analytic dataset.

These findings were tested in sensitivity analyses including overlap-only subsets and outlier influence checks, and alternative inflation handling.

### Ethics approval

Ethics approval was not required as this study involved secondary analysis of publicly available fee schedules and valuation data. The study did not involve recruitment of participants, collection of identifiable personal information, or intervention.

## Results

The master dataset comprised 343 Australian Dental Association (ADA) item codes as shown in Table 1. After excluding codes with DWAU missing or equal to zero and codes with no available comparator fee in the relevant merged series, 260 item codes were included in the merged general analysis and 260 item codes were included in the merged specialist analysis.

**Table 1:** Sample characteristics, fee-source coverage and price-standardisation rules (01 January 2025 baseline).

Component	Summary
Total ADA item codes	343
Codes with DWAU > 0	264
Codes included in merged general series	260
Codes included in merged specialist series	260
Baseline price date	01 January 2025
Inflation rule	ADA and ADF uplifted to 01 January 2025 (CPI factor 1.004323); CDBS and DVA unadjusted (commence 01 January 2025); PHI deflated by 6 months using compounded pro-rata adjustment reduction of 1.014889.
Code coverage by source (n)	ADA general 121; ADA specialist 120; ADF general 336; ADF specialist 337; CDBS 71; DVA general 197; DVA specialist 234; PHI typical prices 40; OHFFSS 83.
**Illustrative fee examples (selected item codes; fees adjusted to 01 January 2025 Australian dollars)**	
**Item code (stream; DWAU)**	**ADA; ADF; CDBS; Private typical price (PHI)**
S415 (Endodontics; 0.370)	ADA 335.37; ADF 286.95; CDBS 242.45; PHI 379.35
S417 (Endodontics; 0.360)	ADA 335.27; ADF 292.56; CDBS 236.15; PHI 368.51
S731 (Prosthodontics; 0.080)	ADA 56.07; ADF 50.60; CDBS 49.90; PHI 53.21
S250 (Periodontics; 0.440)	ADA 253.63; ADF 314.32; CDBS N/A; PHI 301.51

All fees were expressed in 01 January 2025 Australian dollars using the pre-specified standardisation rules. ADA and ADF values (effective 01 July 2024) were uplifted using a CPI factor of 1.004323, while CDBS and DVA fee schedules commencing in 2025 were treated as unadjusted. Private health insurance typical price values were deflated by 6 months to the 01 January 2025 baseline using a compounded pro-rata adjustment reduction of 1.014889.

Comparator coverage varies by source. ADF provided the widest item-code coverage, while ADA survey means and the public schemes (CDBS and DVA) contributed smaller subsets. PHI typical prices were available for only a small subset of 40 item codes.

### Primary findings

Across all included codes, the overall grouped DWAU per dollar (DWAU/$) was 1.589 DWAU per $1000 in the merged general comparator series and 1.130 DWAU per $1000 in the merged specialist comparator series.

Under the merged general comparators, endodontics and prosthodontics returned lower grouped DWAU per dollar than the overall benchmark, while periodontics returned higher grouped DWAU per dollar. Relative to the overall merged general benchmark (1.589 DWAU per $1000), endodontics was 8.66% lower and prosthodontics was 7.46% lower, whereas periodontics was 6.57% higher.

Under the merged specialist comparators, the divergence was most pronounced for endodontics. Relative to the overall merged specialist benchmark (1.130 DWAU per $1000), endodontics was 16.40% lower and periodontics was 7.68% lower, while prosthodontics was close to benchmark (0.98% higher).

### Suggested calibration factors

Suggested stream calibration factors were derived such that, if applied, stream-level grouped DWAU per dollar (DWAU/$) would align to the relevant overall benchmark, as shown in Table 2. Two suggested calibrated schedules were generated within the analytic dataset: one aligned to the merged general benchmark (1.589 DWAU per $1000) and one aligned to the merged specialist benchmark (1.130 DWAU per $1000), the latter supporting derivation of a suggested specialist DWAU rate.

**Table 2:** Stream-level DWAU per dollar results (DWAU per $1000) and pairwise comparisons.

Panel A. Stream estimates relative to the overall benchmark							
Series	Overall benchmark (DWAU^a^ per $1000)		Stream	DWAU^a^ per $1000 (95% CI^b^)	% diff vs benchmark		Suggested calibration factor vs benchmark (95% CI^b^) (Δ%^c^)
Merged general	1.589		Endodontics	1.451 (1.363–1.538)	−8.66%		1.094 (0.990–1.210) (+9.49%)
Merged general	1.589		Periodontics	1.693 (1.588–1.800)	+6.57%		0.938 (0.850–1.037) (−6.16%)
Merged general	1.589		Prosthodontics	1.470 (1.260–1.675)	−7.46%		1.080 (0.981–1.192) (+8.06%)
Merged specialist	1.130		Endodontics	0.945 (0.888–1.004)	−16.40%		1.196 (1.088–1.313) (+19.61%)^d^
Merged specialist	1.130		Periodontics	1.044 (0.902–1.208)	−7.68%		1.083 (0.924–1.266) (+8.32%)
Merged specialist	1.130		Prosthodontics	1.142 (0.965–1.306)	+0.98%		0.990 (0.898–1.099) (−0.97%)
**Panel B. Pairwise stream comparisons**							
**Series**		**Pairwise comparison**				**Difference in DWAU per $1000 (95% CI)**^**b**^	
Merged general		Endodontics–Periodontics				−0.242 (−0.379 to −0.098)^d^	
Merged general		Endodontics–Prosthodontics				−0.019 (−0.253 to 0.205)	
Merged general		Periodontics–Prosthodontics				0.223 (−0.018 to 0.457)	
Merged specialist		Endodontics–Periodontics				−0.099 (−0.271 to 0.050)	
Merged specialist		Endodontics–Prosthodontics				−0.196 (−0.371 to −0.018)^d^	
Merged specialist		Periodontics–Prosthodontics				−0.098 (−0.318 to 0.128)	

Merged general and merged specialist refer to item-level mean comparator fee series constructed from available general and specialist remuneration sources, respectively.

^a^*DWAU* Dental Weighted Activity Unit. DWAU per dollar is expressed as DWAU per $1000.

^b^*CI* confidence interval. Confidence intervals were estimated using non-parametric bootstrap resampling at the item-code level.

^c^Δ indicates the percentage change implied by the suggested calibration factor relative to the existing DWAU value.

^d^Statistically significant at the 5% level.

The range of stream-level DWAU per $1000 across endodontics, periodontics and prosthodontics was 0.242 in the merged general series and 0.196 in the merged specialist series. After applying the suggested calibration factors, stream-level grouped DWAU/$ matched the target benchmarks by construction, meaning the between-stream differences for the adjusted streams were reduced to zero within the analytic dataset.

### Specialist premium

Differences between specialist and general remuneration signals for the same item codes were quantified using a ‘specialist premium’ defined as the merged specialist fee divided by the merged general fee, calculated for codes with both values available (*n* = 260). The overall mean specialist premium was 1.495 (bootstrap 95% confidence interval 1.404–1.602), indicating that specialist comparator fees were, on average, approximately 50 per cent higher than general comparators when mapped to the same ADA item codes. Mean premiums varied by stream, with endodontics 1.498 (95% confidence interval 1.399–1.611), periodontics 1.571 (95% confidence interval 1.412–1.742), and prosthodontics 1.470 (95% confidence interval 1.272–1.761). This pattern indicates that specialist pricing signals are not a uniform uplift from general fees, supporting stream-specific calibration rather than applying a single specialist multiplier across disciplines.

**Fig. 1:**
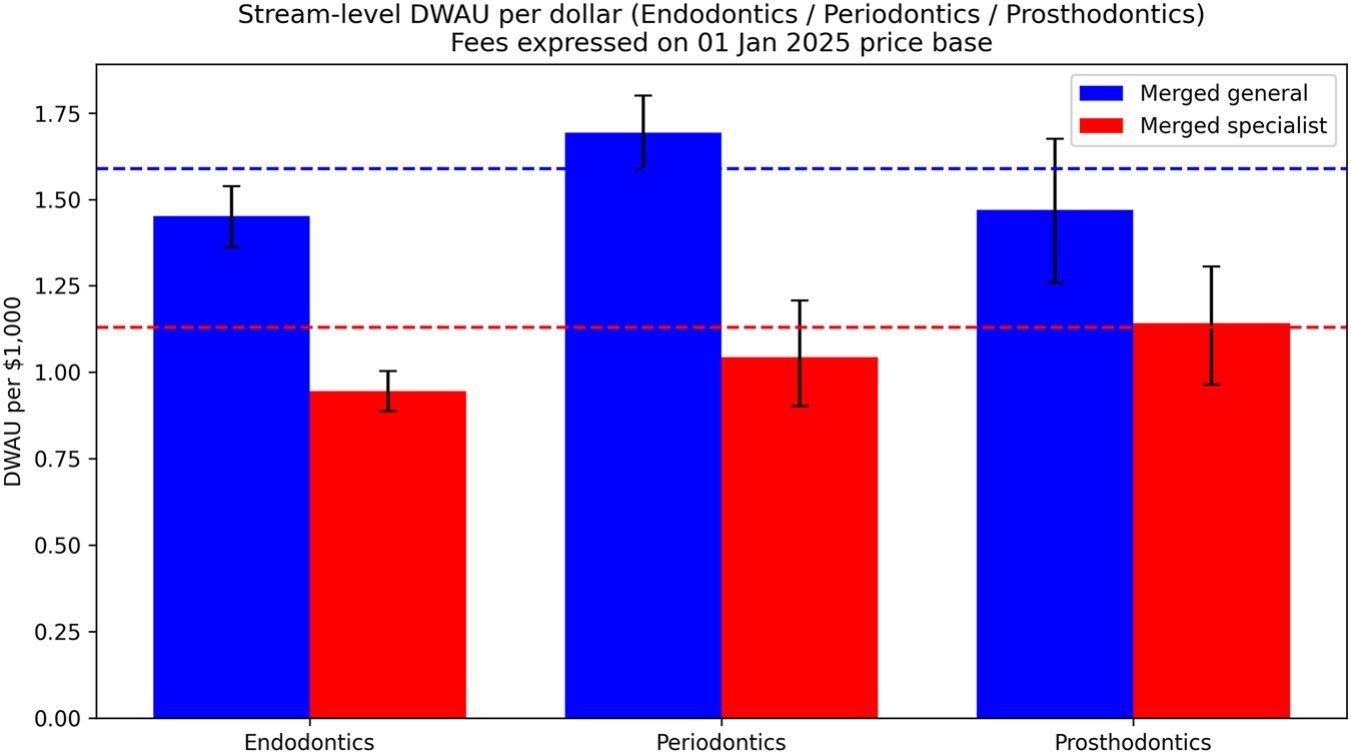
Stream-level DWAU per dollar for endodontics, periodontics and prosthodontics under merged general and merged specialist fee series, with overall benchmark lines (ΣDWAUs ÷ Σfees), shown as DWAU per $1000.

Figure 1 summarises the primary findings that DWAU credited per dollar of external remuneration (DWAU/$) differs by stream. It also shows that the pattern depends on whether general or specialist comparators are used. Error bars show the bootstrap 95% confidence intervals for each stream estimate. Under the merged general series, endodontics and prosthodontics sit below the overall benchmark while periodontics sits above, indicating that these streams receive systematically different DWAU credit per dollar even when assessed against general remuneration signals. Under the merged specialist series, the separation is most pronounced for endodontics, which is 16.40% below the benchmark, while prosthodontics is close to benchmark (0.98% above) and periodontics remains below (7.68% below).

### Sensitivity analysis

Bootstrap uncertainty for grouped DWAU per dollar (DWAU/$) was assessed using non-parametric resampling at the item-code level. Codes were resampled with replacement within each group and grouped DWAU/$ was recalculated as ΣDWAUs ÷ Σfees. These calculations generated 95% confidence intervals. Under specialist comparators, endodontics remained below the overall specialist benchmark (0.945 DWAU per $1000; 95% CI 0.888–1.004 vs benchmark 1.130; 95% CI 1.045–1.209).

A leave-one-source-out (LOSO) analysis was conducted to assess dependence of merged means on any single fee source. Merged means were recalculated after excluding each source (general: ADA, ADF, CDBS, DVA, PHI; specialist: ADA, ADF, DVA, PHI) and grouped DWAU/$ was recalculated. Endodontics remained below the specialist benchmark in all LOSO runs, with deviations ranging from −11.09% (dropping ADF specialist) to −27.06% (dropping ADA specialist). General-series stream differences were stable in overall direction with expected shifts when high-coverage sources were removed.

Inflation and distributional sensitivity checks were undertaken to test price-standardisation choices and influential codes. Analyses using overlap-only subsets and outlier influence checks did not change stream ordering. Repeating analyses without CPI uplift of ADA/ADF fees produced minimal changes (maximum absolute change <0.35%) and did not alter conclusions.

## Discussion

### Principal findings

This study provides a code-level comparison of DWAU values, the activity unit used to measure throughput in Australian public dental services, against multiple Australian remuneration schedules. The main comparison used grouped DWAU per dollar (DWAU/$) as the primary metric, expressed as DWAU per $1000 [2, 14]. In the merged fee comparisons standardised to 01 January 2025 there were consistent between-stream differences in DWAU/$. Endodontic codes showed the largest deviation from the overall benchmark under specialist comparators, with endodontics 16.40% below the specialist benchmark (0.945 vs 1.130 DWAU per $1000). In practical terms, this magnitude implies that a specialist endodontic treatment priced at $1000 is credited with about 0.945 DWAU per $1000 compared with 1.130 at the overall benchmark, indicating a material gap between recorded activity weights and specialist remuneration patterns for endodontics. A simplified example is that a specialist endodontic treatment valued at $1000 corresponds to approximately $836 of value within the DWAU system. Bootstrap uncertainty analyses indicated that this endodontic deviation from the specialist benchmark was statistically significant, whereas periodontic and prosthodontic deviations from the benchmark were not. Periodontics was below the specialist benchmark, whereas prosthodontics was near the benchmark. These findings indicate that the current DWAU schedule does not align with contemporary remuneration signals across dental streams. The proposed calibration outputs should therefore be interpreted as alignment to external remuneration comparators, rather than as a direct estimate of true clinical cost, time, or resource intensity. Weighted activity units function as standardised output measures used to compare activity and support funding based on relative complexity and resource use. Therefore, this misalignment suggests that cross-stream comparisons of output and value may be influenced in part by the weighting system itself rather than solely by underlying differences in clinical workload or resource use [34–37]. The direction of these findings is consistent with the concern that a fixed weighting system may undervalue procedures requiring additional time, materials or skill, so that recorded activity understates the relative input required for some specialist care.

### Differences between specialist streams

The three specialist streams did not diverge from the benchmarks in the same way. Under the merged general series, periodontics sat above the overall benchmark while endodontics and prosthodontics sat below it. Under the merged specialist series, periodontics moved below the benchmark while prosthodontics moved close to it. One possible explanation may be historical. DWAU weights were assigned when the system was established under the 2012 National Partnership Agreement and, in the absence of a scheduled review mechanism, have not been systematically recalibrated since [15, 16]. Weight assignment under time-constrained implementation would not necessarily have captured relative resource intensity evenly across specialist disciplines, and any initial misspecification would persist unchanged while fees continued to evolve, producing the uneven, stream-specific divergence observed here. This explanation is tentative and would be best tested against documentation of the original weight-assignment process and direct time and cost data.

### Interpretation in the Australian funding context

Direct comparisons of DWAUs with external remuneration schedules are limited. The present findings are interpreted in relation to the broader Australian context of fragmented dental funding and multiple co-existing payment models. Australia’s dental funding debate has been described as structurally fragmented with high out-of-pocket spending and a policy divide between public subsidy and private delivery [1, 17]. This fits the broader context for divergence between public valuation tools and remuneration schedules and this supports the proposition that Australian oral health financing remains uneven across public and private settings [1, 38]. The findings show that the current DWAU schedule does not map evenly to contemporary remuneration comparators across streams, which is relevant because weighted activity units are used as standardised measures of output and relative resource use in Australian activity-based funding systems [34, 35, 37].

The analysis is framed as a cross-schedule alignment exercise because Australian dental care operates across multiple remuneration arrangements with different purposes and rules, in a system where patients fund a large share of dental expenditure and cost remains a barrier to care [2]. A valuation unit used for public reporting and commissioning is expected to preserve equivalence when compared against similar remuneration signals. The observed between-stream differences in DWAU per dollar therefore indicate a potential distortion in how recorded activity is credited per dollar of remuneration, which may affect interpretation of output, benchmarking of services and calibration of commissioning assumptions across specialist disciplines. This framing is consistent with wider health financing literature that treats provider payment systems as policy levers that influence incentives and system performance, supporting the use of structured benchmarking rather than assuming a static weighting scale remains aligned over time [39, 40].

### Strengths and limitations

A major strength of this study was the use of multiple Australian comparator schedules rather than reliance on a single benchmark. Additional strengths included the use of Consumer Price Index standardisation to a common price date which allowed for code-level matching across sources. The majority of the data also only required at most 1 year inflation adjustment which limits any potential accumulated errors when implementing this change. Together, these features allowed a detailed examination of whether DWAU values align consistently across streams even with different dated data sources [32, 33]. Several limitations should be highlighted with this research. The analysis was cross-sectional and therefore does not capture how valuation alignment changes over time. Cross-sectional designs are limited in their ability to assess temporal relationships and change processes [41, 42]. Item-code coverage differed across comparator sources, creating missingness and possible composition effects in merged fee estimates. Although sensitivity analyses suggested this effect was small, incomplete coverage may have reduced precision and contributed to only some between-stream contrasts reaching statistical significance. Private health insurance and private specialist fee data were also limited and heterogeneous, reflecting the lack of standardised dental fees across Australian practices. Costs can vary substantially between providers and recent policy debate has highlighted persistent concerns about fee transparency and comparability in specialist charging environments [43].

These constraints reduce precision and mean that the reported estimates should be interpreted as indicative measures of misalignment rather than definitive estimates of true relative resource use.

Consideration was also given to benchmarking DWAU values against government-funded dental schemes in other economies, such as the banded Units of Dental Activity used by the National Health Service in England [44]. A direct code-level comparison was not undertaken because service classification systems, currencies, purchasing arrangements and patient charging rules differ substantially between countries, and no validated mapping exists between ADA item codes and overseas activity classifications. International schemes are therefore drawn on qualitatively when interpreting the findings rather than as quantitative comparators, and the development of formal cross-jurisdictional mappings is identified as a direction for future work [45, 46].

### Equity and access implications

The public health relevance of these findings extends beyond technical schedule design. If some streams are persistently undervalued relative to contemporary remuneration signals then public services may have reduced capacity to sustain those streams. This could be particularly evident in specialist disciplines where delivery depends on scarce workforce capacity, higher treatment complexity and infrastructure that is not easily substituted. Australia’s oral health system is mixed across public, private and non-government providers and limited public capacity has long been linked to barriers for people who cannot readily access private care [47]. Workforce planning evidence also notes shortages in rural and remote areas and highlights that specialist dental care in regional settings is constrained by workforce availability and programme design [48]. Structural undervaluation could plausibly contribute to longer waiting times, tighter referral pathways, or reduced availability of complex care for patients reliant on publicly funded services. Although this study did not measure access outcomes directly, the observed valuation differences provide a mechanism through which funding design can influence service distribution and equity. This interpretation is consistent with Australian evidence that public dental waiting times remain a persistent system issue and that private-sector dominance increases pressure on public access pathways [2, 49]. These considerations are especially important because Australian oral health funding remains heavily reliant on private expenditure. Australian policy commentary has stated that this contributes to unequal access and unmet need, particularly for lower-income groups [17, 50]. Public valuation that lags behind remuneration signals available elsewhere can undermine recruitment and retention in public dental services, because lower remuneration is a commonly reported reason clinicians leave the public sector [51]. This pressure may be greatest for specialist roles that require longer training and have higher practice overheads, potentially worsening maldistribution and reducing access to specialist care for populations reliant on publicly funded services [52, 53].

Equity concerns are further supported by evidence that oral health outcomes are patterned by social disadvantage and remoteness, with poorer oral health in regional and remote populations [47].

### Distinction between remuneration signals and true resource use

Remuneration schedules are indirect signals for valuation, not direct measures of true resource use. In health economics costs are conceptually distinct from prices, charges and reimbursements, because the latter can reflect payer rules, negotiation and market conditions rather than the underlying opportunity cost of resources consumed [54–56]. Schedule fees may therefore embed historical and administrative influences, policy constraints and programme design, in addition to resource cost calculations [35, 54]. A divergence between DWAUs and comparator schedules should not be interpreted as definitive proof that the DWAU weighting system is ‘wrong’ in a strict cost-accounting sense. Instead, it indicates that the current DWAU framework does not map evenly to the remuneration signals operating across contemporary Australian dental funding environments. Benchmarking against private and market-derived schedules should not be read as a proposal that public spending follow private pricing. Market fees are used here as one available signal of contemporary relative value. Other suitable reference points could be publicly costed benchmarks, such as jurisdictional cost collections of the kind used in hospital activity-based funding, and to public fee schedules in comparable jurisdictions, with market-based schedules used as supplementary signals [57].

This distinction is recognised in Australian activity-based funding as weighted activity units and prices are used for funding purposes, while separate costing standards and cost collections are used to estimate efficient cost more directly [35–37, 54, 58]. If DWAUs are used for public output reporting and resource allocation, then substantial and consistent misalignment with comparators remains important because it suggests that the weighting system alters service sustainability and delivery. Future work incorporating direct cost data or treatment duration data would help distinguish whether observed divergence reflects a true clinical resource intensity. Direct measurement approaches, including observation-based costing and time-and-motion methods, are suitable methods for future resources calculations required to deliver care [59–61].

### Policy and workforce relevance

Persistent between-stream differences in DWAU credited per dollar of external remuneration (DWAU/$) imply that a single DWAU schedule can distort how output and value are interpreted across disciplines in the public health system. This aligns with the broader rationale for examining discrepancies across valuation and funding models in specialist dental care, given that remuneration structures in dentistry can influence management and provider behaviour [62]. If some streams receive systematically fewer DWAUs per dollar when benchmarked to remuneration, then activity metrics used for planning may understate the recorded output and resourcing needs of those streams. This is consistent with Australian activity-based funding principles, in which weighted activity measures are used to compare activity and inform funding decisions [34, 35, 58]. Activity-based metrics may understate the resourcing needed for disciplines where remuneration comparators indicate consistently lower DWAU/$. Where public valuation lags remuneration signals, incentives may favour care delivery outside public settings, with potential consequences for service sustainability and patient access. This is consistent with broader health workforce literature showing that pay and financial incentives influence retention and intention to remain in service [63, 64]. It also aligns with research that public-private practice arrangements can affect specialists’ intention to stay in the public sector [65]. Although these outcomes were not directly measured in the present study, they represent system-level implications of persistent valuation misalignment.

### Future research agenda

Future research should examine whether the observed misalignment persists over time using longitudinal comparisons of DWAU and remuneration schedules.

More complete private specialist fee datasets and service-volume data would improve precision and allow weighting by actual activity. Direct measures of resource use, such as time and clinic-level costing studies, would help determine whether revised DWAU values reflect suitable remuneration. These approaches would strengthen the evidence base for modernising public dental valuation frameworks.

### Implications for public health in Australia

The DWAU system and governance should move beyond single-schedule review and adopt a structured programme of cross-schedule benchmarking with scheduled update reviews. A useful Australian comparison is activity-based funding in public hospitals, where the Independent Health and Aged Care Pricing Authority updates its Pricing Framework regularly and publishes transparent pricing methods to support accountability and benchmarking [57]. Applying this logic to public health dentistry would mean that DWAU reviews are undertaken against a structured basket of comparators, in which publicly costed benchmarks and government fee schedules, including those of comparable overseas public systems, included. The inclusion of market-derived schedules would also be used as supplementary signals rather than as targets. This is consistent with wider health financing literature recognising provider payment as a health-system lever that creates economic signals and influences service delivery [39, 40]. International experiences also show the risks of static or poorly aligned payment systems. In the United Kingdom, repeated reviews of the NHS dental contract have described it as unfit for purpose and linked it to persistent access problems and ongoing calls for reform [45, 46]. A DWAU update cycle would therefore strengthen transparency and reduce the risk that misalignment accumulates over time.

These findings support whole-schedule recalibration rather than only incremental code-by-code edits. Stream-level differences in DWAU per dollar suggest that misalignment may be structural rather than driven by a small number of outliers. Lower DWAU per dollar indicates fewer credited DWAUs for a stream, which can distort planning metrics. Hospital pricing provides a precedent for revising activity weights as costs and models of care change [57]. Payment systems shape incentives and service organisation which means that distortions in weighting may require schedule-level correction rather than local adjustments alone [39, 66]. Reducing this distortion may improve output metrics and support more appropriate resource allocation, particularly for endodontics.

Revising the relative weights for individual item codes or through suggested stream-level calibration factors would change how activity is credited between disciplines without necessarily changing total funding. Changing the funding attached to each DWAU would alter resourcing across the board but could not correct between-stream misalignment. The present findings concern primarily the relative weights and a uniform change in the price per DWAU would leave this relative distortion in place.

There is a rationale for developing a specialist DWAU schedule or specialist multiplier to reflect additional specialist inputs and resource intensity. A strong comparator is the Resource-Based Relative Value Scale used for physician payment in the United States. This system links payment relativities to pre-measured resource inputs and has structured review processes for updating [67–70]. Applying this logic to dentistry would allow for a specialist-adjusted DWAU framework which would better acknowledge the expertise, time, equipment and infrastructure required for specialist procedures. A specialist DWAU rate could support commissioning of specialist services, reduce the risk of under-provision of complex care in public settings, and improve workforce planning for endodontic, periodontal and prosthodontic activity.

Internationally, publicly funded dental activity is remunerated through diverse mechanisms, including banded activity units in England, resource-based relative value scales in the United States, and blended fee-for-service, capitation and salaried arrangements elsewhere [44, 67]. Weighting and payment schedules that are not periodically recalibrated against measured inputs or contemporary comparators tend to drift, and this drift manifests as access problems, provider withdrawal from public schemes and distorted activity reporting. The wider conclusion for dental funding policy is therefore not specific to Australia and any jurisdiction that uses fixed activity weights to fund or report dental care requires a transparent, scheduled review mechanism and cross-jurisdictional benchmarking of how different types of dental activity are valued offers an underused check on local schedule drift [39, 40, 45, 46, 67].

## Conclusions

This study showed that current DWAU values are not aligned with contemporary Australian remuneration signals across dental streams. Systematic differences were identified in DWAU credited per dollar of external remuneration (DWAU/$) in both merged general and merged specialist comparator series. The largest divergence was identified in endodontics under specialist comparators, where DWAU/$ was lowest relative to the overall benchmark. The suggested stream-specific calibration factors indicated that this between-stream dispersion could be removed within the study dataset, were the factors to be applied. This supports the argument that the present weighting schedule may not fully align with contemporary remuneration signals across general and specialist dental care. These findings illustrate a lesson relevant to publicly funded dental systems internationally that fixed activity weights can drift out of alignment over time, and periodic benchmarking should be a routine feature of dental funding governance.

## Data Availability

The datasets used and analysed during the current study are available from the corresponding author on reasonable request.
